# Protein-templated copper nanoclusters for fluorimetric determination of human serum albumin

**DOI:** 10.1007/s00604-021-04764-7

**Published:** 2021-03-08

**Authors:** Mariagrazia Lettieri, Pasquale Palladino, Simona Scarano, Maria Minunni

**Affiliations:** Department of Chemistry “Ugo Schiff”, University of Florence, 50019 Sesto Fiorentino, FI Italy

**Keywords:** Copper nanoclusters, Human serum albumin, Fluorescence, Label-free assay

## Abstract

Copper nanoclusters (CuNCs) are attractive for their unique optical properties, providing sensitive fluorescent detection of several kinds of targets even in complex matrices. Their ability in growing on suitable protein and nucleic acid templates make CuNCs efficient optical reporters to be exploited in bioanalysis. In this work, we report the specific and sensitive determination of human serum albumin (HSA) in human serum (HS) and urine via CuNCs fluorescence. HSA is the most abundant protein in plasma, and plays a key role in the early diagnosis of serious pathological conditions such as albuminuria and albuminemia. Recently, HSA has become clinically central also as a biomarker to assess severity, progression, and prognosis of various cancers. We report the controlled and reproducible growth of CuNCs directly on the target analyte, HSA, which results in a fine dose-dependent fluorescent emission at 405 nm. The protocol is optimized in water, and then applied to serum and urine specimens, without matrix pretreatment. The method linearly responds within the whole concentration of clinical interest, with a sensitivity of 1.8 ± 0.1 × 10^−3^ g L^−1^ and 0.62 ± 0.03 × 10^−3^ g L^−1^ in serum and urine, respectively, and excellent reproducibility (CV_av_% ca. 3% for both). The assay is designed to have a single protocol working for both matrices, with recovery of 95% (HS) and 96% (urine). The stability of the fluorescence after CuNCs formation was tested over 3 days, displaying good results (yet higher in urine than in serum).

**Supplementary Information:**

The online version contains supplementary material available at 10.1007/s00604-021-04764-7.

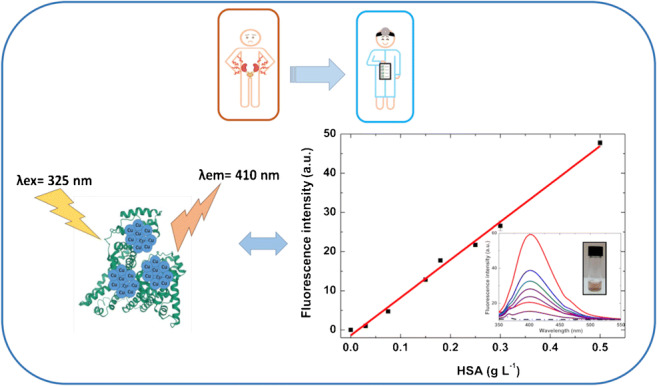

## Introduction

Human serum albumin (HSA), the most abundant protein in human blood (35–50 g L^−1^), is a multi-domain protein able to bind different endogenous and exogenous macromolecules. Therefore, the key role of HSA consists in carrying many biomolecules and, consequently, in modulating oncotic blood pressure [1]. In addition, HSA is a notable biomarker of many diseases such as cancer [2], liver disorders [3] (i.e., cirrhosis or hepatitis), rheumatoid arthritis [4], diabetes, hypertension, kidney disease, and cardiovascular diseases [5, 6]. In particular, low HSA concentration in human serum and high HSA values in urine denote two important pathological states known, respectively, as albuminemia and albuminuria [7, 8]. As a whole, the accurate determination of HSA in different biological specimens plays a critical role both from a diagnostic and a prognostic point of view. In the past, precipitation-based methods were commonly applied to detect albumin [9]. Presently, a large number of analytical methods (i.e., immunochemical, dye-binding, chromatographic, spectroscopic, and electrophoresis-based methods) are reported in literature for the accurate estimation of HSA [10–12]. For HSA detection in blood, electrophoretic technique represents the first choice [13], whereas immunonephelometry and immunoturbidimetry are preferred for HSA determination in urine samples [14, 15]. There is a lack, therefore, of a method allowing HSA determination in these two specimens through the same procedure, i.e., in a matrix-independent manner. Moreover, most of the analytical methods proposed so far, taken together, suffer from some disadvantages because are often expensive, time-consuming, and laborious, requiring in some cases (e.g., electrophoretic investigations) many pre-analytical steps and the use of synthetic dyes. In this framework, here we present a low cost and rapid method for the accurate and selective determination of HSA, both in human serum and urine, without the need for any sample pre-treatment (except dilution for human serum). The outstanding properties of copper nanoclusters (CuNCs) [16] were exploited to develop an assay able to directly detect albumin-templated CuNCs by fluorescence. As far as we know, this is the first report on the use of CuNCs for the selective albumin detection for clinical diagnostics. Differently from metallic nanoparticles, widely employed in bioanalytics, metallic nanoclusters are still poorly explored for such applications. However, in recent years, the unique fluorescent properties of CuNCs, such as high quantum yield, photostability, and large Stokes shifts, enabled their frequent use in numerous bioanalytical assays [17, 18]. In addition, remarkable features as low toxicity and good biocompatibility allow the use of CuNCs as fluorophores for biolabeling and bioimaging [19], representing an alternative to the quantum dots and organic dyes. However, two works referred about CuNCs and HSA [20, 21]. In the first, bilirubin is quantified in urine and human blood samples by the quenching induced by the analyte upon its interaction with red fluorescent CuNCs synthesized by using HSA as a template [20]. In the second, Chen et al. quantified HSA in plasma by following the reduction of fluorescence of poly(thymine)-templated copper nanoparticles (CuNPs) due to HSA that inhibits the CuNPs formation [21]. Differently from above reported turn-off fluorescence strategies, here we report for the first time that HSA-templated CuNCs are able to directly detect and quantify HSA itself by a turn-on strategy that exploits the selective growth of CuNCs on the albumin template. The optimized experimental conditions only require the addition of Cu(II) and NaOH aliquots to the serum/urine sample, followed by 1–3 h of incubation at 55 °C. The blue-emitting HSA-templated CuNCs allow to detect HSA with high sensitivity, selectivity, and reproducibility in serum and urine specimens without any pretreatment, except for a 1:300 dilution of serum in water. Serum dilution allowed to process serum and urine within the same calibration range but could be tailored on the basis of specific testing requirements. The excellent analytical performances of the developed assay represent a valid alternative to current screening techniques, paving the route toward an effective and innovative method for HSA detection in clinical diagnostics.

## Materials and methods

### Chemicals and reagents

Copper sulfate (CuSO_4_) and sodium hydroxide (NaOH) were from Thermo Fisher Scientific (Parma, Italy). Human serum albumin (HSA), human serum from male AB (HS), and human IgG were purchased from Merck (Milan, Italy). Artificial urine (AU) was from LCTech GmbH (Obertaufkirchen, Germany). High Select™ HSA/Immunoglobulins (IgG, IgA, IgM, IgD, and IgE) Depletion Mini Spin Columns used for selectivity studies were from Thermo Fisher Scientific (Rodano, MI). The columns were used by following the manufacturer’s instructions.

### Instrumentation and optical measurements

HSA-CuNCs were synthesized in water, serum, or urine samples, by incubation at 55 °C (Thermomixer comfort, Eppendorf, VWR International, Milan). Fluorescence experiments were performed with Spectrofluorometer FP-6500 (Jasco, Easton, PA, USA), and absorbance measurements were recorded by using Thermo Scientific™ Evolution™ 201/220 UV-Visible Spectrophotometer (Rodano, MI). Data management was performed by using OriginLab (Origin Pro 8.5.1) software. In order to define the proper excitation wavelength, λ_ex_ were scanned from 325 to 450 nm, by using an interval of 10 nm. Experimental conditions: 1.5 g L^−1^ HSA-CuNCs, wavelength range 200–650 nm, integration time 60 s, spectral bandwidth 1 nm, scan speed 100 nm min^−1^. This step allowed us to establish the best conditions for fluorescence measurements, i.e., λ_ex_ = 325 nm and λ_em_ = 405 nm (emission bandwidth 5 nm, excitation bandwidth 5 nm, data pitch 1 nm, scanning speed 100 nm min^−1^, sensitivity low). The same operative conditions were used for water, serum, and urine samples.

### Assay protocol for HSA determination

HSA-templated CuNCs were synthesized starting from the protocol proposed by Goswami et al. [22], with some modifications, as reported in the Electronic Supporting Material (ESM), Scheme SI 1. The fluorescent HSA-templated CuNCs do not require any purification step. All the measurements were performed at 25 °C in triplicates, at least. The same protocol is used for water, serum, and urine samples, except for 1:300 serum dilution in water, which permits it to work within the same calibration range for both the matrices. The linear trend of the assay in HS was first evaluated by the standard addition method, since it naturally contains physiological HSA (ca. 35–50 g L^−1^). To this aim, a series of equal HS aliquots were prepared, adding to each a different concentration of HSA (standard in water, concentration range 0–0.50 g L ^−1^). Subsequently, the protocol proceeded as described in ESM. The fluorescence emission intensity of the samples was measured in triplicate both just after the reaction ended (intraday variation) and for the three consecutive days (inter-day standard deviation), by subjecting the samples to a daily freeze/thaw cycle before fluorescence measurement.

## Results and discussions

### CuNCs synthesis and optical behavior

**Fig. 1:**
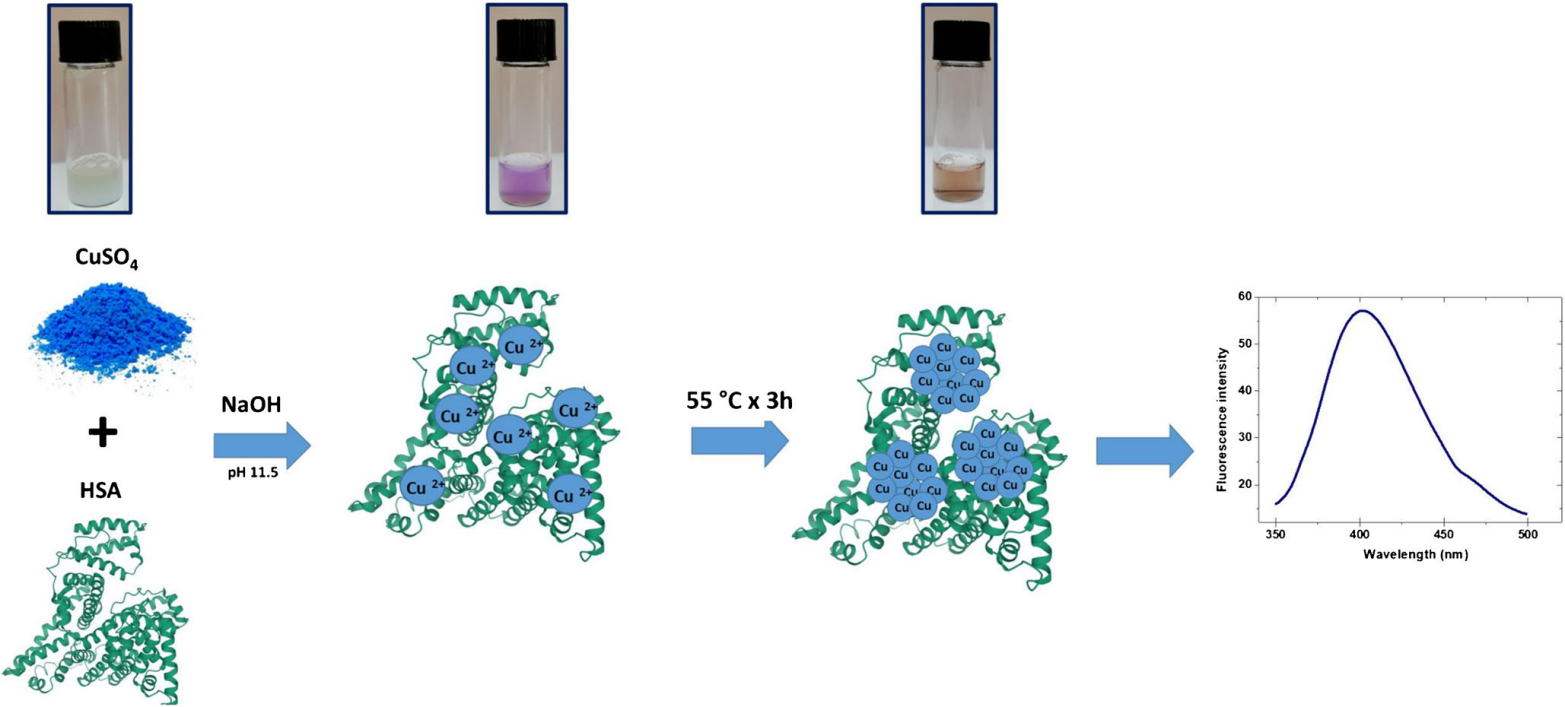
Schematic illustration of the experimental steps involved in HSA-CuNCs synthesis

The experimental procedure optimized to synthesize HSA-CuNCs gave highly photoluminescent nanoclusters in an easy, quick, and cheap manner (Fig. 1 and Scheme SI 1). Advantageously, this protocol does not require purification steps or the use of reducing agents nor toxic substances as reported by other procedures [23, 24]. This simplifies the detection of compounds of interest in complex biological matrices, such as urine, human serum, and human blood. The mechanism of nanoclusters formation involves principally amine, thiol, and carboxyl groups of HSA that act as template and trigger the formation and stabilization of the nanoclusters by using -NH_2_ and –COOH groups for the coordination of Cu(II), while the –SH groups allow the reduction mediated by alkaline pH of copper ions to metallic Cu atoms, which aggregate in stable nanoclusters.

The absorption spectra of HSA-CuNCs obtained in water show a main peak at 280 nm, due to aromatic amino acids, and a second peak, weaker, at 325 nm, characteristic of copper nanoclusters, as previously reported [22] (ESM, Fig. SI 1). The absence of the characteristic localized surface plasmon resonance (LSPR) band of copper nanoparticles between 500 and 600 nm [25] confirms the effective formation of nanoclusters while avoiding the presence of bigger nanoparticles. The maximum intensity of fluorescence emission (Fig. SI 2A) was obtained by using λ_ex_ = 325 nm, with the emission peak centered at 405 nm, as reported elsewhere [22]. Accordingly, when the fluorescence at 405 nm was examined, the excitation spectrum of HSA-CuNCs exhibited a sharp peak at 325 nm, and both show a clear dose-response trend as a function of HSA concentration in solution (Fig. SI 2B). For this reason, all fluorescence measurements were performed employing λ_ex_ = 325 nm and λ_em=_ 405 nm. The smaller shoulder at about 375 nm, particularly evident at low analyte concentrations, is due to the Raman scatter peak, which depends on solvent and excitation wavelength [26].

### Optimization of parameters for HSA detection

The photoluminescent properties of CuNCs strongly depend on the medium pH and, in particular, an alkaline pH is necessary for their formation. At pH 7.0, Cu(II) is reduced to Cu(I), which binds the protein structure to form the metalloprotein complex. Subsequently, when the pH is raised close to 12.0, copper ions are further reduced to metallic copper (Cu(0)) that leads to the initiation of the nucleation and growth of the clusters [22].To optimize the CuNCs formation, the pH influence was carefully evaluated by preparing several aqueous HSA-CuNCs solutions at pH ranging from 9.0 to 13.0. The maximum luminescence signal was reached at pH 11.5 (see Fig. SI 3), presumably due to the enhancement of the reducing properties of albumin thiol groups, which promote the effective formation of copper nanoclusters. This pH value was thus kept for all the following experiments. We also investigated the ability of the method in giving valuable responses in the shortest possible time with the aim to improve the current protocols in diagnosing and monitoring hypoalbuminemic and microalbuminueric patients. At this purpose, the Cu(II)/HSA mixtures were incubated from 1 to 7 h at 55 °C, monitoring the fluorescence signal evolution. As shown in Fig. SI 4, the highest fluorescent emission was obtained after 3 h, reaching a plateau stage for a longer incubation time, followed by a decrease of the signal around 7 h. Therefore, 3 h was chosen as the best incubation time for CuNCs formation, which is shorter than reaction times from 5 to 8 h previously reported for HSA-CuNCs formation [27, 28]. However, these results may foresee the possibility of shortening the incubation time to ca. 1 h, reinforcing the real applicability of the method in clinical settings. We also explored the possible influence of the stirring speed (200, 500, 1000, and 1400 rpm (rpm)) during the formation of the clusters, finding that this parameter does not affect the final CuNCs fluorescence intensity (data not shown). The CuNCs obtained with the optimized protocol show excellent fluorescence stability up to 4 months of storage at room temperature and/or after multiple freezing/thawing steps (data not shown).

### Sensitive detection of human serum albumin

#### HSA detection in water solution

**Fig. 2:**
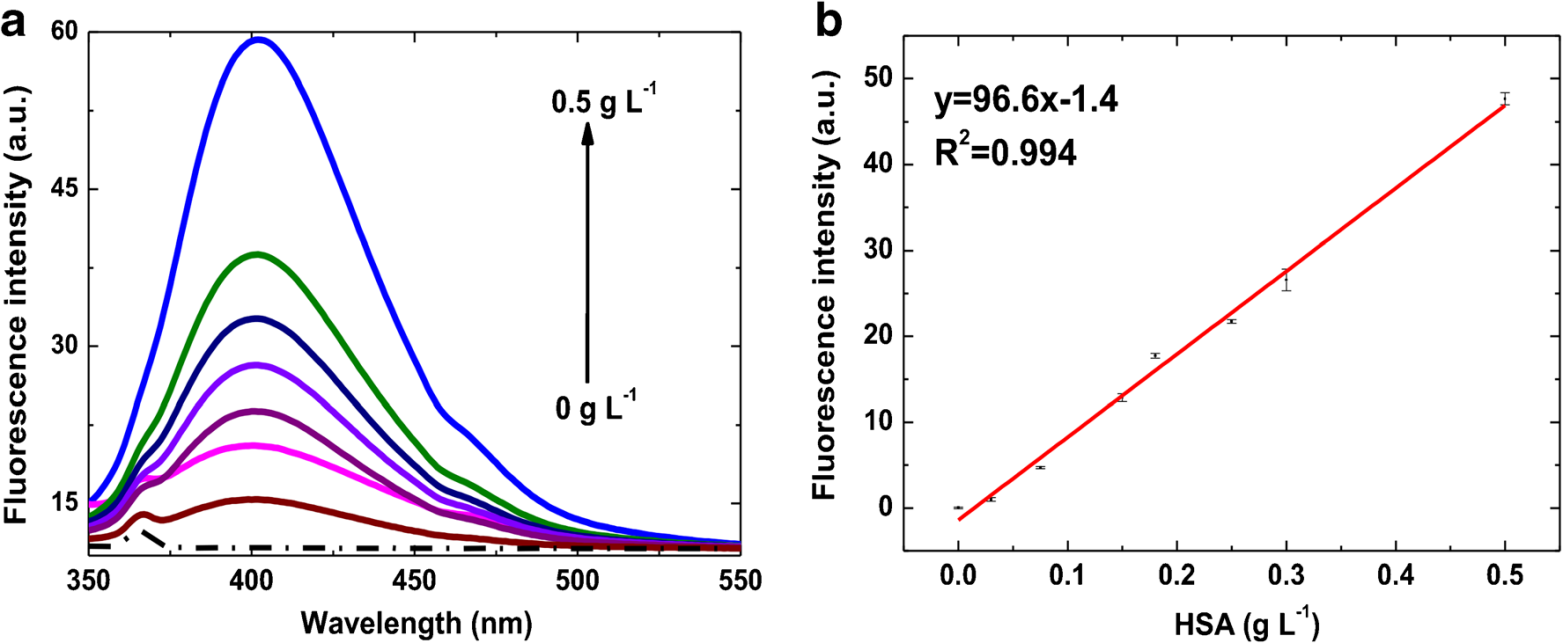
HSA-CuNCs fluorescence in water: (**a**) Emission spectra at different HSA concentrations. Dashed line is the blank sample (H_2_O). (**b**) Calibration plot of CuNCs fluorescence intensity at 405 nm versus HSA concentration after blank subtraction. The error bars represent the triplicate measurements (intraday standard deviation)

Preliminary studies were carried out in Milli-Q water in order to study the calibrator in ideal conditions. To this aim, the fluorescence spectra of HSA-CuNCs formed in solution at different concentrations of HSA were recorded (Fig. 2a), showing a clear dose-response. The intensity of the fluorescence signal, i.e., the peak centered at 405 nm (with a minor shoulder at 460 nm), was considered to construct the standard HSA calibration within the range 0.03–1.50 g L^−1^ (Fig. 2b). It shows a very well-correlated (*R*^2^ = 0.994) linear response (*y* = 96.6 x-1.4) up to 0.5 g L^−1^ (Fig. 2b), with a slight deviation at higher concentrations of HSA (data not shown). The limit of detection (LOD) obtained was 2.48 ± 0.07 × 10^−3^ g L^−1^ (LOD = 3*SD_blank_/slope), with an average coefficient of variability (CV_av_%) of 5%. The inter-day stability of HSA-CuNCs in water was also investigated. After CuNCs formation, the samples were stored at −20 °C, and then subjected to a 3-day freeze/thaw cycles, giving the fluorescence data reported in Fig. SI 5A. The overall variability, expressed as CV_av_% over all the data collected over the 3 days (7 concentration points for each calibration, each point in triplicate, see Fig. SI 5B), resulted in 9%, confirming the good reproducibility of the measurements despite the freeze/thaw cycles the samples were subjected to. The overall recovery of fluorescence intensity at the second and the third day resulted in 92% and 70%, respectively, compared to the intraday results.

#### HSA detection in human serum

**Fig. 3:**
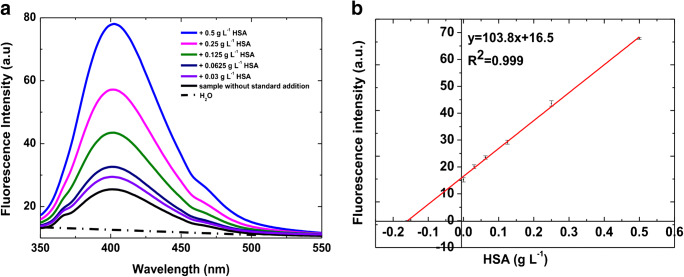
HSA-CuNCs in human serum: (**a**) Emission spectra at different HSA concentrations. Dashed line is the blank sample (HSA-depleted serum). (**b**) Calibration plot corresponding to the quantitative determination of HSA by using the standard addition method. Fluorescence intensity values, in the calibration plot, were obtained by the subtraction of blank fluorescence signal (HSA-depleted serum). The error bars represent the intraday standard deviation calculated on three replicates

The blue emitting HSA-CuNCs were successfully applied to albumin quantification in untreated human serum (except 1:300 water dilution), where HSA values lower than 35 g L^−1^ define a pathological state known as hypoalbuminemia [7]. The patients suffering from analbuminemia, in which the HSA level is less than 1 g L^−1^, are rare [7]. However, both hypoalbuminemia and analbuminemia may be signs of severe liver, kidney, or gastrointestinal diseases, as well as cancer evolution [29–31], and all require the accurate determination of albumin. Albumin is the most abundant protein in human serum with physiological concentrations generally between 35 and 50 g L^−1^ [7]. Therefore, to obtain its absolute estimation, we first applied the standard addition method to assess the linear dose-response trend in the matrix. Due to the high albumin concentration in human serum with respect to our calibration range, a 1:300 dilution of the serum sample is preliminary performed. Then, known increasing concentrations of standard HSA were spiked (0.03–0.50 g L^−1^ final concentration range) to six aliquots of the same serum sample. CuNCs formation was finally carried out simultaneously on all the aliquots following the protocol optimized in water, and the fluorescence responses were recorded (Fig. 3). Also, in this case, we confirmed the presence of a well-defined peak centered at 405 nm (with a minor shoulder at 460 nm). As evidenced in water, the overall optical response is thus related to the endogenous HSA in the sample plus the contribution of the spiked standard HSA. An excellent linear correlation (*R*^2^ = 0.999) allowed us to fit the data and extrapolate the concentration of serum HSA, which resulted in 48.0 ± 1.8 g L^−1^. The CV_av_% resulted in 3%, highlighting the excellent reproducibility of the proposed assay.

The inter-day variability of the method was estimated over 3 days, as previously performed in water, to assess the possibility of storing the processed serum samples until the analysis (Fig. SI 6A). In this case, the overall CV_av_% averaged on all the collected data resulted significantly higher than that obtained in water (19%), although the most marked effect of signal decrease was observed between the first and second day. Due to the complexity of the matrix, the observed progressive decrease could be reasonably ascribed to some serum components that interfere with fluorescence stability over time. However, data up to 0.25 g L^−1^ of the second and the third day show recovery values on all the concentrations of 80% and 73%, respectively. This could indicate a stabilization of the fluorescence after the first 24 h and/or the first freeze/thaw cycle that should be investigated for longer times. The selectivity of the CuNCs-based fluorescent assay was first tested by considering that the protein portion of human serum other than albumin is predominantly composed by immunoglobulins. Therefore, we reproduced the assay on immunoglobulin solutions to assess the absence of their contribution in the final fluorescent signal. Fig. SI 7A shows that the fluorescent spectra recorded in the IgG solutions are comparable to a blank solution. Moreover, we have excluded possible interferences due to other matrix components by using separation columns to remove HSA and immunoglobulins from samples. Also, such depleted samples have given negligible response (see Fig. SI 7B), confirming the selectivity of the method.

#### HSA detection in urine

**Fig. 4:**
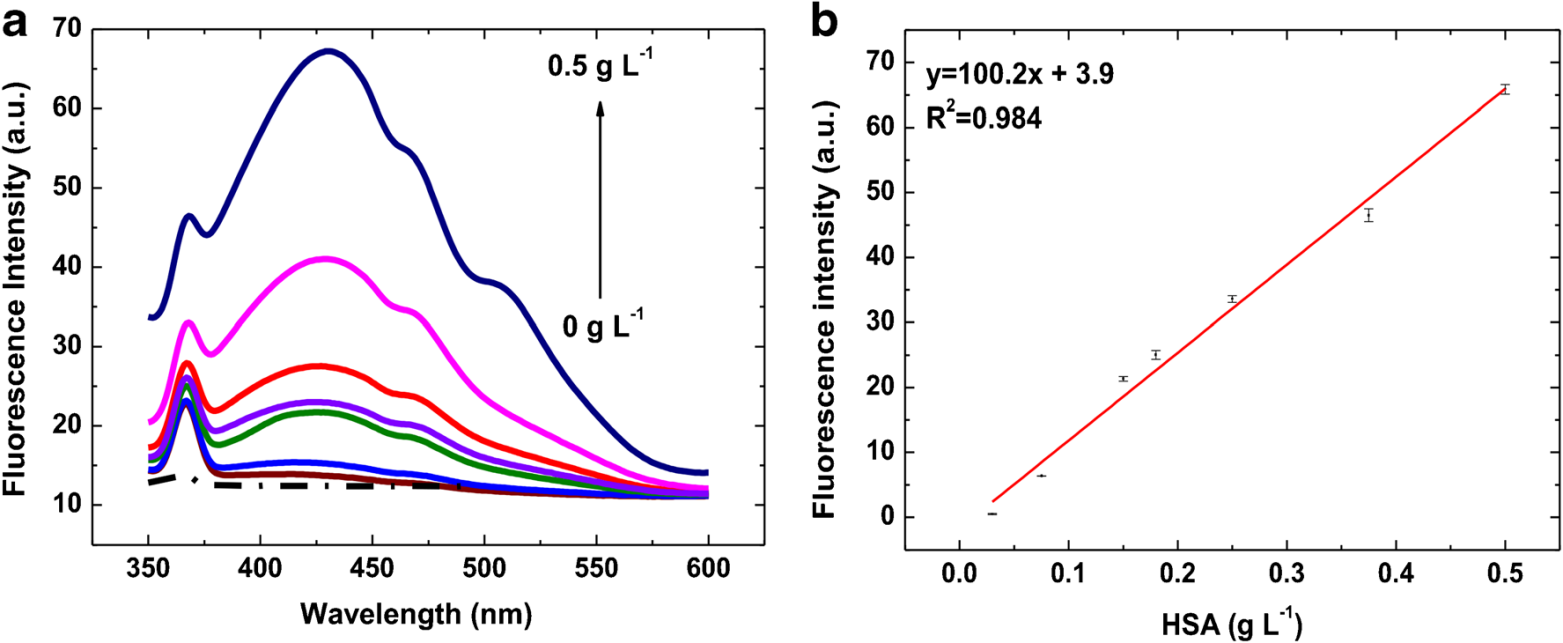
HSA-CuNCs in artificial urine: (**a**) Emission spectra at different HSA concentrations. Dashed line is the blank sample (unspiked urine). (**b**) Calibration plot of standard HSA in artificial urine. Fluorescence intensity values were obtained by the subtraction of the blank (unspiked urine). The error bars represent the triplicate measurements (intra-day standard deviation)

Albuminuria consists in release of a large quantity of albumin in urine as a consequence of kidney damage. The mild form of albuminuria is known as microalbuminuria and is characterized by low HSA values in urine, i.e., 0.02–0.2 g L^−1^, whereas higher values are associated with the severe condition called macroalbuminuria [5]. Cardiovascular and kidney diseases, such as hypertension and diabetes mellitus, are closely related to micro and macroalbuminuria [5, 6]. To demonstrate the applicability of the method also to urine matrix, artificial urine samples were added with increasing standard HSA concentrations, and the CuNCs growth was conducted as above for water and serum. Differently from serum, urine does not contain physiological HSA; therefore, a classical dose-response calibration was carried out by testing urine samples fortified with HSA in the range 0.01–0.50 g L^−1^ (Fig. 4). Figure 4 a displays the intensity evolution of CuNCs fluorescence upon HSA increase. Differently from water and serum, spectra recorded in urine show two minor peaks at wavelengths higher than the main emission peak (Fig. 4a). Moreover, the latter appears red-shifted to ca. 425 nm, likely due to the fact that in this case the matrix is not subjected to dilution in water as above reported for serum. The calibration curve, described by the equation *y* = 100.2*x* + 3.9, correlates with a *R*^2^ value of 0.984 giving a LOD of 0.62 ± 0.03 × 10^−3^ g L^−1^ and a CV_av_% = 3%. The best linear correlation was obtained up to 0.50 g L^−1^, and after that, a loss of linearity is clearly visible (data not shown). Considering the clinical range of interest, the assay showed its ability in detecting with high precision and accuracy even microalbuminuria condition. The inter-day standard deviations display an averaged calibration curve with a very similar trend and a CV_av_% of 10% (Fig. SI 8). The recovery efficiency, inferred by comparing the fluorescence responses obtained over the 3 days, resulted in 98% and 90% for the second and the third day, respectively.

#### Performance of the assay in serum and urine

**Fig. 5:**
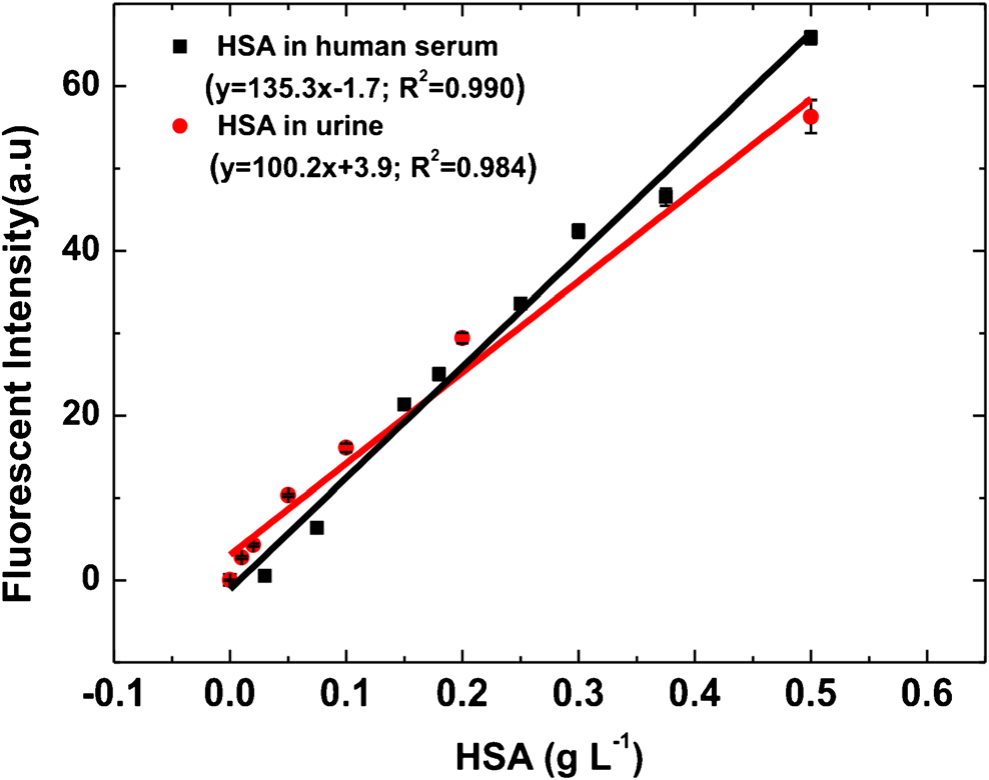
Analytical performance of HSA detection assay in human serum (black line) and urine (red line). The respective blanks (HSA-depleted serum for HS, and unspiked urine for urine samples) were subtracted to the fluorescence signals. Fluorescence values are reported as the mean value ± intraday standard deviation) calculated on three replicates

To reinforce the validity of the developed method, we compared the dose-response curves obtained in both the matrices by testing a serial dilution of serum and urine containing the same HSA content (serum and urine absorbance and fluorescence spectra are reported in Fig. SI 9). In the case of HS, we diluted a sample of known HSA concentration to 0.50 g L^−1^; to directly compare the responses, an undiluted aliquot of urine was spiked with the same HSA concentration, and the same serial dilutions were performed and tested for both the matrices. As reported in Fig. 5, a very good overlapping of the dynamic ranges over the tested conditions is found within the whole range of concentration. In these conditions, the limit of detection in serum can be also directly extrapolated, resulting in 1.8 ± 0.1 × 10^−3^ g L^−1^, with a CV_av_% = 3%. This result enables HSA detection at concentrations lower than those achieved by reference techniques [32, 33]. In addition, recovery has been calculated for all the urine and human serum spiked samples by comparison with the calibration curve of HSA in Milli-Q water. A recovery value of 95% for human serum and of 96% for urine was obtained.

#### Assay performances compared to other nanomaterials-based methods

The CuNCs-based assay here developed was finally compared to other nanomaterial-based methods reported in literature for HSA detection (Table 1). Despite the declared LODs referred to standard solutions display better performances in all the cases, no information about the performances obtained in human serum and urine is reported (except for Huang et al. [34]), and none of them shows the possibility of applying the assay to both the matrices by the same protocol. Since from a clinical point of view the lowest concentration of interest for HSA in urine (in serum the interest goes to the relative variation of physiological levels) is around 0.02 g L^−1^, the reported LODs play a marginal role in the evaluation of a realistic application of the method for clinical purposes. Moreover, some detection methods, such as SERS and ELSD, are far from being considered simple since they require multiple synthesis steps for the nanomaterials involved in the protocol and expensive instrumentation. Finally, a CuNCs-based platform is cheaper than gold- and silver-based nanomaterials, since Cu(II) salts are less expensive than the relative Au(III) and Ag(I) ones. In this framework, the assay here developed allows to achieve a step forward in its application in clinical settings.

**Table 1:** Nanomaterial-based methods for the determination of HSA

*Probe*	*Method*	*LOD*	*Specificity*	*Matrix*	*References*
HSA-templated copper nanoclusters (CuNCs)	Spectrofluorimetry	2.48 ± 0.07 × 10^−3^ g L^−1^ (in H_2_O); 1.8 ± 0.1 × 10^−3^ g L^−1^ (in human serum) 0.62 ± 0.03 × 10^−3^ g L^−1^ (in urine)	Yes	Artificial urine Human serum	This work
Poly(thymine)-templated copper nanoparticles (CuNPs)	Spectrofluorimetry	8.2 × 10^−11^ g L^−1^ (in buffer solution)	Yes	Human serum	[21]
Gold nanoparticles (AuNPs)	Colorimetry	9.3 × 10^−5^ g L^−1^ (in urine)	Yes	Artificial urine	[34]
Gold “pearl necklace” (Au PNNs)	Surface-enhanced Raman scattering (SERS)	4.6 × 10^−6^ g L^−1^ (in buffer solution)	Yes	Artificial urine	[35]
Nanoporous gold (NPG)	SERS	0.1 × 10^−9^ g L^−1^ (in buffer solution)	–	–	[36]
Gold nanorods (AuNR)	Surface plasmon resonance (SPR)	–	–	Artificial urine	[37]
AuNPs	Evaporative light scattering detection (ELSD)	21.0 ng (in buffer solution)	–	Human serum	[38]
Silver nanoparticles (AgNPs)	Localized surface plasmon resonance (LSPR)	1.0 × 10^−6^ g L^−1^ (in buffer solution)	Yes	Urine	[39]

## Conclusions

A novel CuNCs-based assay for the fluorescent detection of HSA in urine and human serum is reported. Both the matrices are successfully analyzed by the same protocol, without significant pretreatment (except for a 1:300 dilution of serum in water), and high selectivity. The whole protocol is performed within 3 h, but it could be further reduced down to 1 h, in line with clinical requirements. The sensitivity of the CuNCs-based assay resulted higher than other diagnostic methods, i.e., 0.03–1.00 g L^−1^ in serum, and 0.01–0.75 g L^−1^ in urine, with excellent intraday variability (3%). Despite at this development stage, we demonstrated its applicability to reinforced matrices, and we foresee that the method could be further validated for its application in clinical diagnostics with improved accuracy, shorter times, and very low costs with respect to current available assays. To the best of our knowledge, this is the first example of an assay for HSA able to work in serum and urine without the need of a matrix-dependent protocol and very similar analytical performances.

## Supplementary information

ESM 1 (DOCX 364 kb)
