# Supplementary material for: Protein-templated copper nanoclusters for fluorimetric determination of human serum albumin
Source: Mikrochim Acta. 2021 Mar 8;188(4):116. doi: 10.1007/s00604-021-04764-7 (PMC7940276; doi:10.1007/s00604-021-04764-7)
Supplement: Supplementary file 1 — (DOCX 364 kb) [file 604_2021_4764_MOESM1_ESM.docx]

**Electronic Supporting Material**

**Protein-templated copper nanoclusters for fluorimetric determination of human serum albumin**

**Mariagrazia Lettieri, Pasquale Palladino, Simona Scarano*, Maria Minunni**

*Department of Chemistry “Ugo Schiff”, University of Florence, 50019, Sesto Fiorentino, FI, Italy*

[**simona.scarano@unifi.it*](mailto:*simona.scarano@unifi.it)

**Materials and methods**

**Artificial urine composition:** (pH 6.6 ± 0.1): 25.00 g L^-1^ urea, 9.00 g L^-1^sodium chloride, 2.50 g L^-1^potassium dihydrogen orthophosphate, 2.50 g L^-1^disodium hydrogen orthophosphate anhydrous, 3.00 g L^-1^sodium sulphite hydrated, 3.00 g L^-1^ ammonium chloride and 2.00 g L^-1^ creatinine.

**CuNCs synthesis:** 1 mL of 0.02 mol L^-1^ CuSO_4_ water solution is added to 5 mL of standard HSA (15 g L^-1^ in water or matrix), obtaining a sudden turbid-light blue coloring. The solution is then stirred for 2 min at room temperature and then adjusted at pH 11.5 (30 µL, 5 mol L^-1^ NaOH), changing the color in limpid-purple. Subsequently, the sample is stirred at 500 rpm for 3 hours at 55 °C, showing finally a brown color.


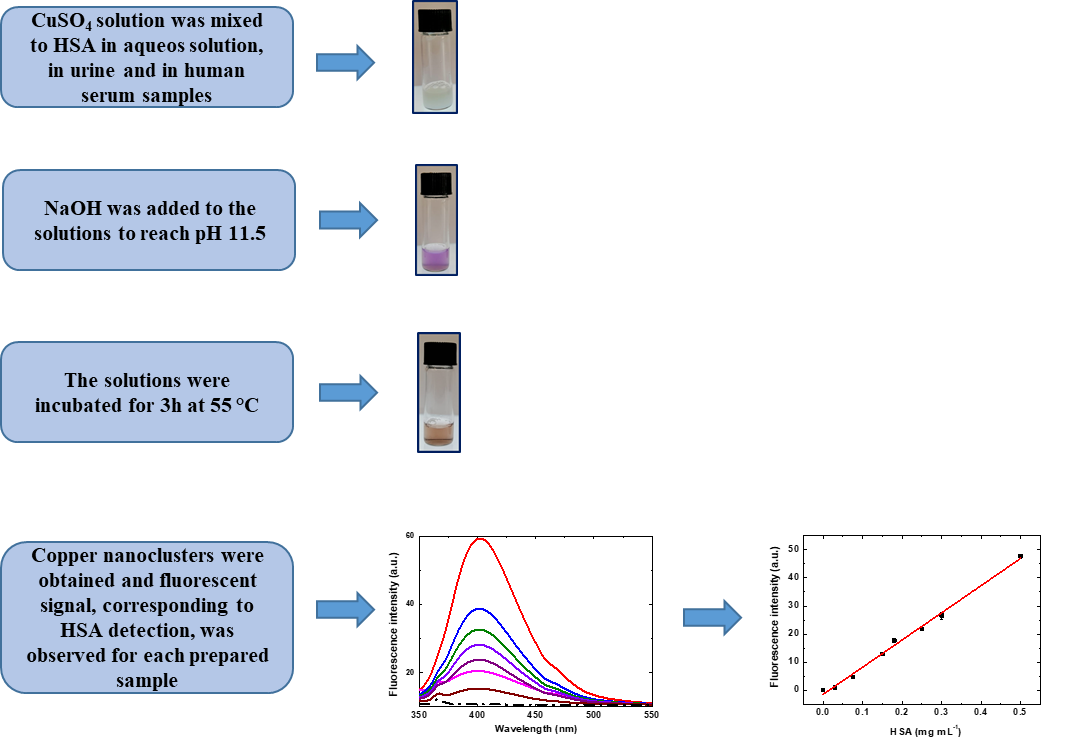


Scheme SI 1. Representation of the whole experimental procedure adopted to detect HSA in different biological matrices.

**Separation columns protocol:** 100 µL of diluted human serum (1:300) were added to the columns and incubated for 10 minutes at room temperature. The columns were centrifuged at 1000 x g for 2 minutes. The filtrate was collected to be processed as a ‘blank’ sample (*i.e.* not containing HSA) and subjected to fluorescence measurements.

**
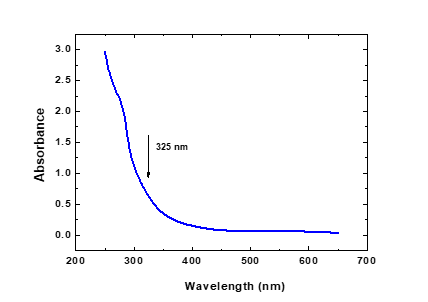
**

**Fig. SI 1** UV-vis absorption spectrum of HSA-CuNCs in water solution.

**
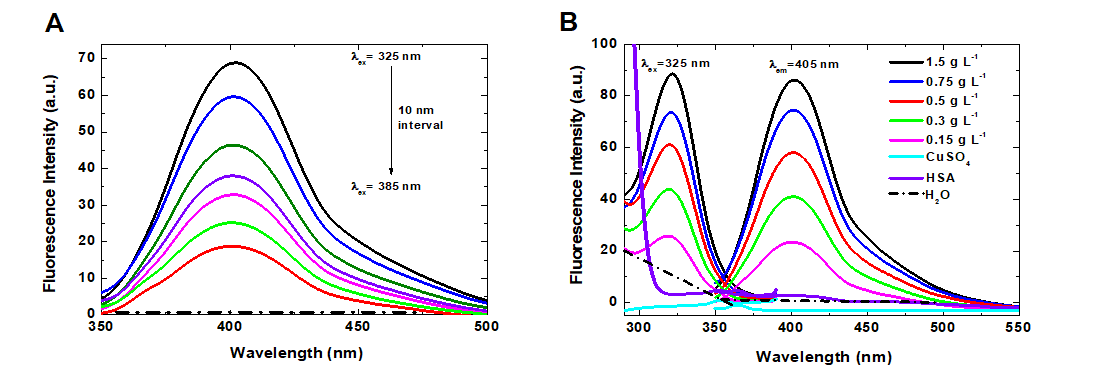
Fig. SI 2** HSA-CuNCs in water: A) emission spectra at different excitation wavelengths. B) emission and excitation spectra at different HSA concentrations. Dashed line is the blank sample (H_2_O).


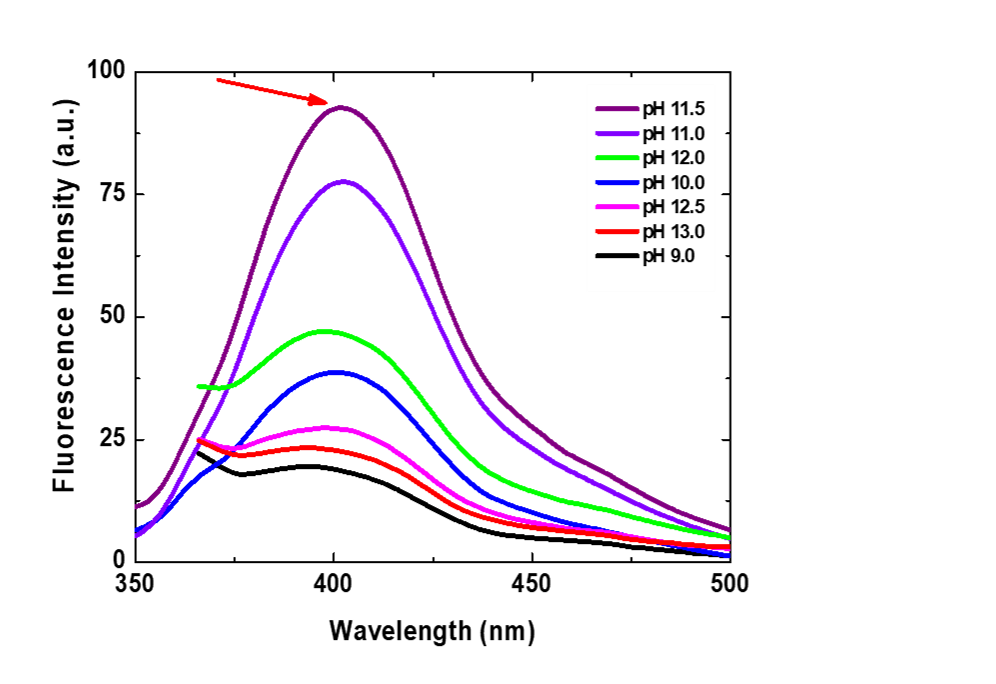


**Fig. SI 3** Emission spectra of HSA-CuNCs in water solutions at different pH values, ranging from 9.0 to 13.0. The arrow indicates the maximum fluorescent signal corresponding to the pH adopted during the CuNCs synthesis.


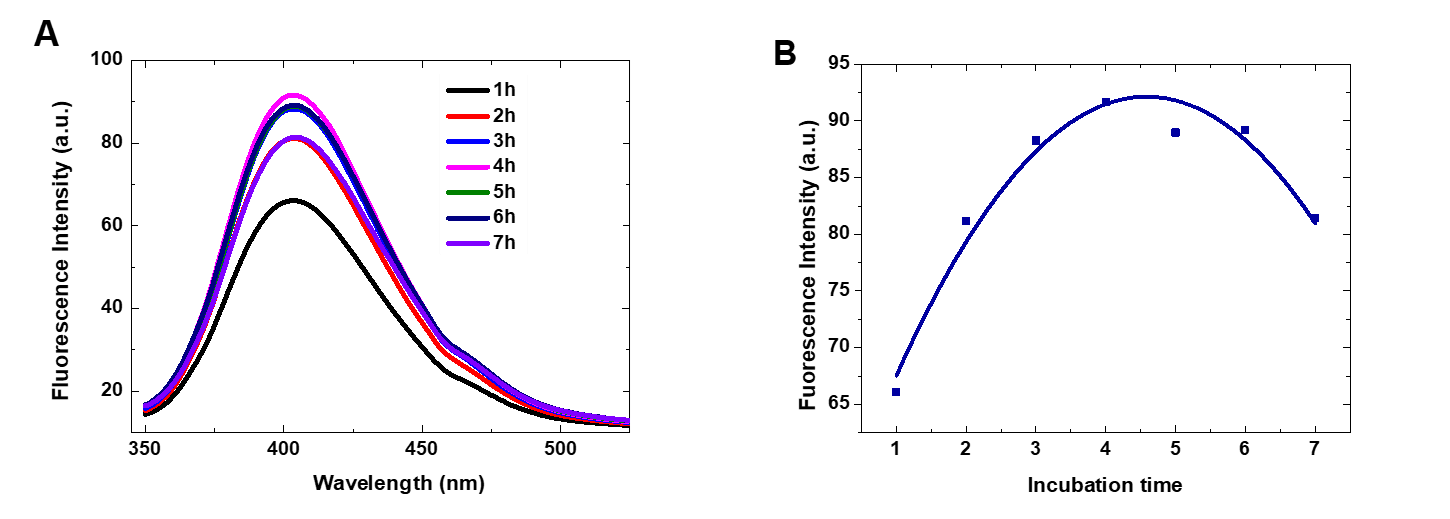


**Fig. SI 4** Time evolution of the fluorescence emission spectra during the CuNCs formation in water solution.


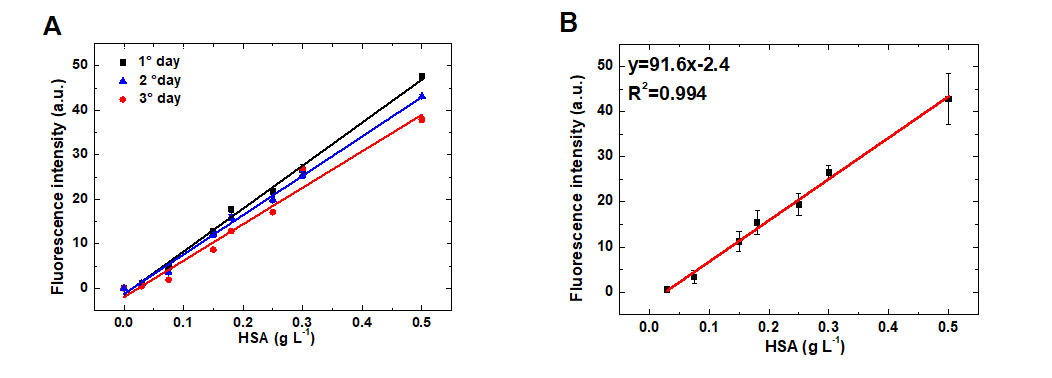


**Fig. SI 5 A)** Calibration plots of HSA determination in *milli Q water*, over three different days (error bars represent the intraday triplicate measurements). **B)** Cumulative inter-day calibration curve corresponding to the average of the fluorescence measurements carried out over 3 different days (error bars represent the interday triplicate measurements). The relative calibration plots equation and R^2^ are reported in **Table SI 1**. Fluorescence intensity values were obtained by the subtraction of blank fluorescence signal (H_2_O).

**Table SI 1** Calibration plots equation and R^2^ values of calibration plots reported in Figure SI 5.

|  |  | **Calibration plot equation** | **R^2^ values** |
| --- | --- | --- | --- |
|  |  |  |  |
| **Figure S4 A** | **1° day (black)** | y=96.6x-1.4 | 0.994 |
|  | **2° day (blue)** | y=88.5x-1.2 | 0.994 |
|  | **3° day (red)** | y=82.0x-2.0 | 0.970 |
| **Figure S4 B** | **Cumulative 1°-3° days** | y=91.6x-2.4 | 0.994 |

**
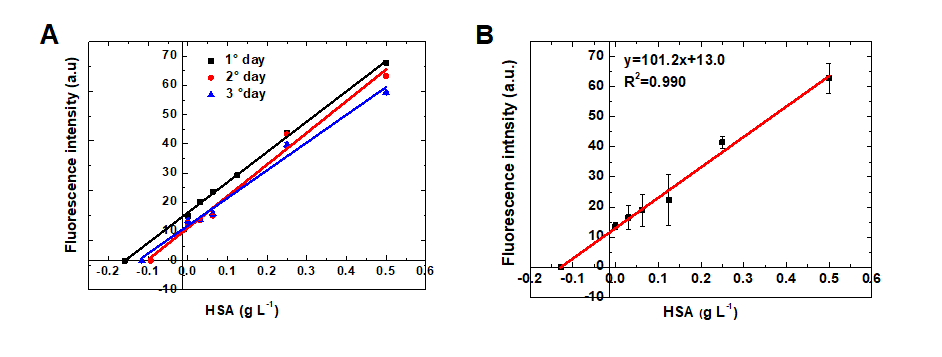
Fig. SI 6** **A)** Calibration plots of HSA determination in *human serum,* over three different days (error bars represent the intraday triplicate measurements). **B)** Cumulative inter-day calibration curve corresponding to the average of the fluorescence measurements performed in 3 different days (error bars represent the interday triplicate measurements). The relative calibration plot equation and R^2^ are reported in **Table SI 2**. Fluorescence intensity values were obtained by the subtraction of blank fluorescence signal (HSA-depleted serum).

**Table SI 2** Calibration plots equation and R^2^ values of calibration plots reported in Figure SI 6.

|  |  | **Calibration plot equation** | **R^2^ values** |
| --- | --- | --- | --- |
|  |  |  |  |
|  | **1° day (black)** | y=103.8x+16.5 | 0.999 |
| **Figure S5 A** | **2° day (red)** | y=108.6x+11.2 | 0.982 |
|  | **3° day (blue)** | y=94.9x+12.0 | 0.985 |
| **Figure S5 B** | **Cumulative 1°-3° days** | y=101.2x+13.0 | 0.990 |


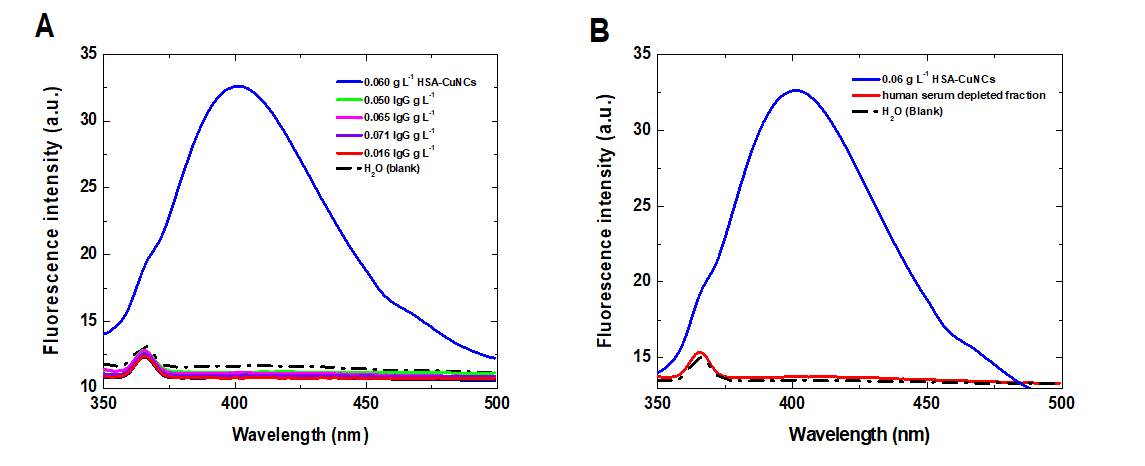


**Fig. SI 7** A) Fluorescent spectra of different IgG concentrations in comparison with HSA-CuNCs fluorescent emission signal (blue line). B) Spectrum of samples obtained after separation of diluted human serum (1:300) on High Select™ HSA/Immunoglobulin Depletion Mini Spin Columns. The depleted fraction of human serum (red line), in which HSA and immunoglobulins were removed, did not show fluorescence at 405 nm characteristic of HSA-CuNCs solution (blue line), appearing superimposable to milli Q water (blank) emission response (black dashed line).


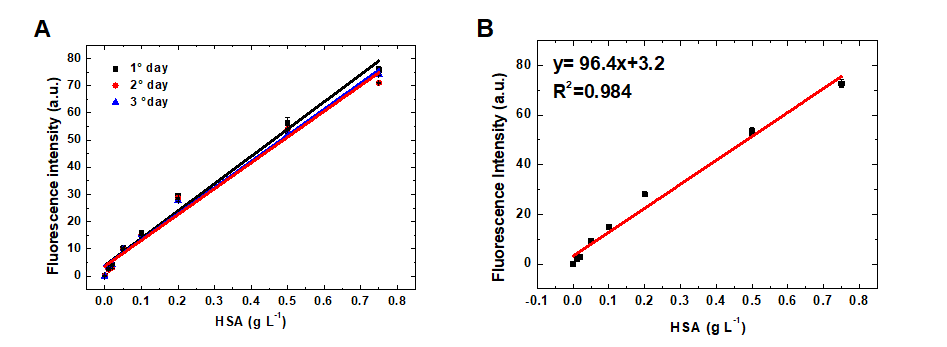


**Fig. SI 8 A)** Calibration plots corresponding of HSA determination in *urine*, over three different days (error bars represent the intraday triplicate measurements). **B)** Cumulative inter-day calibration curve corresponding to the average of the fluorescence measurements performed over 3 different days (error bars represent the interday triplicate measurements). The relative linear equations and R^2^ values are reported in **Table SI 3**. Fluorescence intensity values were obtained by the subtraction of blank fluorescence signal (unspiked urine).

**Table SI 3** Calibration plots equation and R^2^ values of calibration plots reported in Figure SI 8.

|  | **Day** | **Calibration plot equation** | **R^2^ values** |
| --- | --- | --- | --- |
|  |  |  |  |
|  | **1° (black)** | y=100.2x+3.9 | 0.984 |
| **Figure S8 A** | **2° (red)** | y=94.9x+3.7 | 0.980 |
|  | **3° (blue)** | y=96.2x+3.7 | 0.988 |
| **Figure S8 B** | **Cumulative 3 days** | y=94.4x+3.2 | 0.984 |


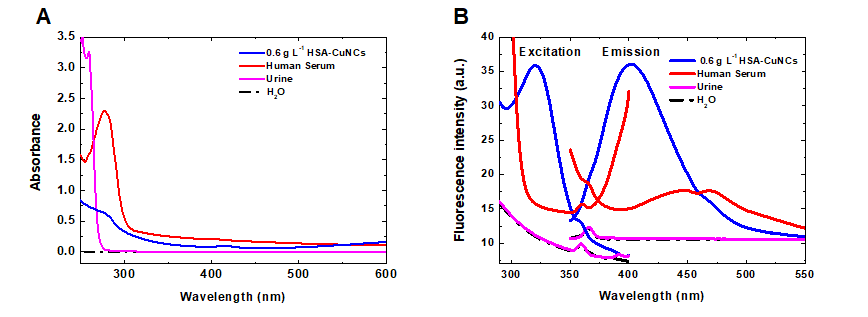


**Fig. SI 9 A)** Absorbance spectra of: HSA-CuNCs (blue line) where the peak at 320 nm (due to CuNCs formation, see Section 3.1) and 280 nm (due to aromatic compounds) were observed; human serum (red line, diluted 1:300) where the peak due to aromatic amino acid at 280 nm appeared; urine (pink line) and water (black dashed line). **B)** Fluorescence spectra of: HSA-CuNCs (blue line) in which the emission and the excitation peak are clearly illustrated; human serum (red line, diluted 1:300); urine (pink line) and water (black dashed line). Note as the analyzed matrices, urine and human serum, does not interfere under UV light used as excitation wavelength (325 nm).
